# Guidelines for cell-type heterogeneity quantification based on a comparative analysis of reference-free DNA methylation deconvolution software

**DOI:** 10.1186/s12859-019-3307-2

**Published:** 2020-01-13

**Authors:** Clémentine Decamps, Florian Privé, Raphael Bacher, Daniel Jost, Arthur Waguet, HADACA consortium, Sophie Achard, Elise Amblard, Raphael Bacher, Fabian Bergmann, Michael Blum, Yuna Blum, Guillaume Bottaz-Bosson, Lucile Broseus, Florent Chuffart, Clémentine Decamps, Emilie Devijver, Ghislain Durif, Vassili Feofanov, Eugene Andres Houseman, Melina Gallopin, Paulina Jedynak, Vincent Jonchere, Ellen van de Geer, Basile Jumentier, Tony Kaoma, Eugene Lurie, Pavlo Lutsik, Julia Markowski, Anna Melnykova, Jane Merlevede, Petr Nazarov, Ngoc Ha Nguyen, Olga Permiakova, Florian Privé, Magali Richard, Matthieu Rolland, Michael Scherer, Yannick Spill, Eugene Andres Houseman, Eugene Lurie, Pavlo Lutsik, Aleksandar Milosavljevic, Michael Scherer, Michael G. B. Blum, Magali Richard

**Affiliations:** 1 Laboratory TIMC-IMAG, UMR 5525, Univ. Grenoble Alpes, CNRS, F-38700 Grenoble, France; 2 HeAlth DAta ChAllenge (HADACA) collaboration Group, Univ. Grenoble Alpes, CNRS, F-38700 Grenoble, France; 3 Independent Statistical Consultant, La Center, WA USA; 4 Bioinformatics Research Laboratory, Molecular and Human Genetics Department, Baylor College of Medicine, Houston, TX USA; 5 Division of Cancer Epigenomics, German Cancer Research Center (DKFZ), Heidelberg, Germany; 6 Department of Genetics/Epigenetics, Saarland University, 66123 Saarbruecken, Germany

**Keywords:** Cell heterogeneity, Deconvolution, DNA methylation, Epigenetics, Matrix factorization, R package/pipeline

## Abstract

**Background:**

Cell-type heterogeneity of tumors is a key factor in tumor progression and response to chemotherapy. Tumor cell-type heterogeneity, defined as the proportion of the various cell-types in a tumor, can be inferred from DNA methylation of surgical specimens. However, confounding factors known to associate with methylation values, such as age and sex, complicate accurate inference of cell-type proportions. While reference-free algorithms have been developed to infer cell-type proportions from DNA methylation, a comparative evaluation of the performance of these methods is still lacking.

**Results:**

Here we use simulations to evaluate several computational pipelines based on the software packages MeDeCom, EDec, and RefFreeEWAS. We identify that accounting for confounders, feature selection, and the choice of the number of estimated cell types are critical steps for inferring cell-type proportions. We find that removal of methylation probes which are correlated with confounder variables reduces the error of inference by 30–35%, and that selection of cell-type informative probes has similar effect. We show that Cattell’s rule based on the scree plot is a powerful tool to determine the number of cell-types. Once the pre-processing steps are achieved, the three deconvolution methods provide comparable results. We observe that all the algorithms’ performance improves when inter-sample variation of cell-type proportions is large or when the number of available samples is large. We find that under specific circumstances the methods are sensitive to the initialization method, suggesting that averaging different solutions or optimizing initialization is an avenue for future research.

**Conclusion:**

Based on the lessons learned, to facilitate pipeline validation and catalyze further pipeline improvement by the community, we develop a benchmark pipeline for inference of cell-type proportions and implement it in the R package *medepir*.

## Background

Since the development of high-throughput sequencing technologies, cancer research has focused on characterizing genetic and epigenetic changes that contribute to the disease. However, these studies often neglect the fact that tumors are constituted of cells with different identities and origins (cell heterogeneity) [1]. Quantification of tumor heterogeneity is of utmost interest as multiple components of a tumor are key factors in tumor progression and response to chemotherapy [1].

Advanced microdissection techniques to isolate a population of interest from heterogeneous clinical tissue samples are still not feasible in daily practice (too complicated and costly). An alternative is to rely on computational deconvolution methods that infer cell-type composition. Recently, several “reference-free” algorithms have been proposed to estimate tumor cell-type heterogeneity from global DNA methylation profiling of surgical specimens [2–4]. Indeed, DNA methylation is a stable molecular marker with a cell type-specific profile dynamically acquired during cell differentiation [5] and thus provides valuable information for cell-type heterogeneity characterization and quantification. The “reference-free” algorithms are labelled as such because they do not require a priori information about DNA methylation profiles of cell types found within tumors: they directly infer them from DNA methylation samples using computational methods. While not requiring the a priori reference information, some algorithms are designed to use such information when available (e.g., [2–4]). In the absence of reference information, confounding factors affecting methylation values, such as age and sex, can potentially influence the inference of cell-type proportions. Moreover, the heuristics that are used to estimate the number of underlying cell types may differ between each deconvolution method and the sensitivity of the methods to such variability remains uncharacterized. Therefore, there is an urgent need to comprehensively characterize the current analysis pipelines, identify key features influencing their performance and provide benchmarks and recommendations to guide the application and further development of pipelines that quantify cell-type heterogeneity from reference-free DNA methylation samples [6].

Methods correcting for cell-type heterogeneity have already been compared for their statistical power to detect significant associations between epigenetic variation and biological traits [7, 8]. When associating epigenetic variation to phenotypic traits (Epigenome Wide Association Studies, EWAS), cell-type proportions are considered as confounding factors, their inference is not the main objective, but rather an intermediate step that can contribute to reducing false positive associations [9–12]. In contrast, we here compare reference-free deconvolution methods with the estimation of cell-type proportions as the main objective, as they are directly related to tumorigenesis [1]. This objective excludes several software packages from our comparison that instead return latent or surrogate variables, which are not interpretable in terms of cell-type proportions [7].

We compare three software packages that infer cell type proportions based on methylation data: RefFreeEWAS, MeDeCom and EDec [2–4]. For our comparisons, we rely on simulations where real methylation profiles of different cell types are mixed in differing proportions. While some of the methods include series of steps that may be considered a pipeline, the simulations focus on comparing the core deconvolution step shared by all the three methods (e.g., Stage 1 of EDec) that solves a convolution equation that contains two key variables: (i) the cell-type proportions within the samples, and (ii) the average methylation profiles of constituent cell types. The main outcome of this core deconvolution step are estimates of cell-type proportions and of the methylation profiles of constituent cell types, which are needed to characterize the constituent cell types and quantify tumor heterogeneity. Because accurate references for cell-type specific methylation profiles are sparse, especially for solid tissues and cancer cell types, we further assume that reference data for constituent cell-types is not available, which excludes reference-based methods from our comparative analysis [13, 14].

We here evaluate key factors affecting performance of deconvolution pipelines. We examine to what extent cell-type proportions can be accurately inferred when accounting for measured confounding factors. We determine how feature selection impacts algorithms’ performance at inferring cell-type proportions. We study performances variability according to the randomly selected initialization of local optimization involved in solving deconvolution equation. We also test several methods for selecting appropriate number of constituent cell types and ask how sensitive the results are to the variation in cell type number. Based on these, we provide general guidelines for the development of reference-free deconvolution pipelines and define a benchmark pipeline to catalyze further application and improvement of reference-free deconvolution methods.

## Results

### Evaluation of computational frameworks to estimate cell type composition

We apply MeDeCom, EDec (Stage 1, the core deconvolution step), and RefFreeEWAS to estimate heterogeneity within simulated tumorous tissues (Lutsik et al. 2017; Onuchic et al. 2016; Houseman, Molitor, and Marsit 2014). Simulations are encoded in a matrix D of size MxN, where M represents the number of CpG probes and N represents the number of samples. All these software packages perform various types of non-negative matrix factorization to infer cell type proportions (matrix A of size KxN, with K as the putative number of cell types) and cell type-specific methylation profiles (matrix T of size MxK) by solving D = TA, or rather by minimizing, under various constraints (that vary between the three tested algorithms), the error term: ∥*D* − *TA*∥_2_ (see Material and Methods). We simulate D with 5 cell types (K = 5): 2 cancer-like cells (lung epithelial and mesenchymal), healthy epithelial cells (lung epithelial), immune cells (T lymphocytes), and stromal cells (fibroblasts). These simulations mainly depend on a parameter α_0_, which controls the diversity of the generated samples (see Material and Methods): When α_0_ is small (~ 1), the simulated proportions of the K cell-types are diverse among samples and as α_0_ increases, the variability decreases to the point at which proportions are the same for all samples. Finally, we simulate the effect of confounding factors on these mixtures by using a regression model of methylation data computed from real lung cancer clinical datasets (Additional file 1: Figure S1, see Material and Methods for details).

**Fig. 1:**
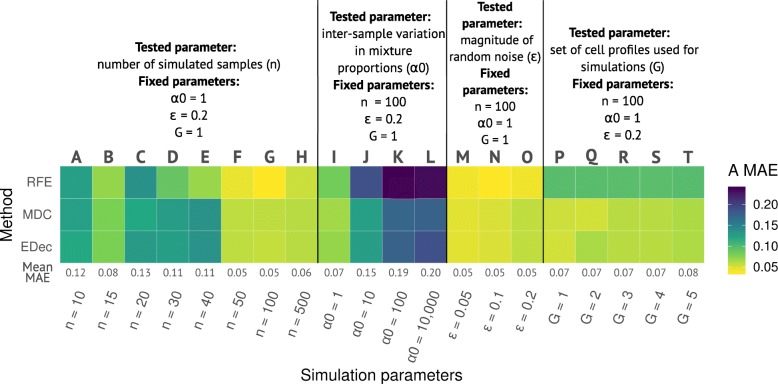
Performance of the 3 deconvolution methods for different parameter settings. Heatmap of method performance (`A MAE`: Mean Absolute Error on estimated A, the matrix of cell proportions). RFE stands for RefFreeEWAS, MDC for MeDeCom and EDec for EDec stage 1. All algorithms were run on 10 D matrices corresponding to 10 different realizations of the random *ε*- controlled process on one D matrix computed from one simulated A matrix, each time, with the following parameters n (number of samples), α_0_ (inter-sample variation in mixture proportion), *ε* (magnitude of random noise applied on D) and G (the cell profiles used for simulations). Mean MAE corresponds to the average error of the three methods (computed for each parameter set). A random A matrix was used for testing the effect of G1 and G2, another random A matrix was used for testing the effect of *ε* magnitude. Testing the effect of n and α_0_ required independent simulation of A each time. As a consequence, the four simulations corresponding to the set of parameters *n* = 100, α_0_ = 1, *ε* = 0.2, G = 1 have different results, because these simulations are based on different randomly simulated A matrices (see Fig. 7 for a systematic analysis of performance variation according to the random simulations of A)

To evaluate the methods performance, we use Mean Absolute Error (MAE, see Material and Methods) as a metric to compare inferred individual cell type proportions to the ground truth. First, we tested the effect of altering four simulation parameters on the methods performance (Fig. 1 and Additional file 1: Figure S1, 1) the number of simulated samples (N, ranging from 10 to 500, 2) the inter-sample variation in mixture proportions (α_0_, from 1 to 10,000, 3) the magnitude of random noise added to the mixture component (*ε*, from 0.05 to 0.2, 4) the set of K cells profiles used to simulate complex tissues (termed as the cell background, G, which includes all specificities related to the cell profile establishment, such as the donor genetic background or the method used to generate the profile: cell lines or primary cells) (see Material and Methods and Additional file 2: Table S4 for details). As expected, increasing the sample size (Additional file 1: Figure S2) improves the performance of all methods (Fig. 1 columns A to H). Increasing inter-sample proportion variability also substantially improves performance of all methods (Fig. 1 columns I to L). Average error (mean error across the three methods) is 0.074 (α_0_ = 1, column I) when inter-sample variation is large, increases to 0.147 (α_0_ = 10, column J) when variation is moderate, and reaches 0.194 (α_0_ = 100, column K) when variation is almost zero (Fig. 1 and Additional file 1: Figure S3). By contrast, the performances of the three methods are neither sensitive to changes of the cell background (Fig. 1 columns P to T and Additional file 1: Figure S4) nor to variations in the magnitude of the random noise applied during simulations (Fig. 1 columns M to O).

In this first direct comparison, the three deconvolution methods account for all 23,381 probes corresponding to a subset of the Illumina 27 k and 450 k DNA methylation probes, with no specific filtering. To run the algorithms, we used the following functions and parameters: RefFreeEWAS::RefFreeCellMix (5 cell types, 9 iterations), EDec::run_edec_stage_1 (5 cell types, all probes kept as informative loci, maximum iterations = 2000), and MeDeCom::runMeDeCom (5 cell types, lambdas in 0, 0.00001, 0.0001, 0.001, 0.01, 0.1), maximum iterations = 300, 10 random initializations, number of cross-validation folds = 10). Under these not-optimized conditions (i.e. with no pre-processing steps), we observe that all methods provide comparable performance, each algorithm performing best under specific conditions and parameter settings. RefFreeEwas performs best for 9 out of 20 different parameter settings, MeDeCom for 8, and EDec for 3 conditions (lowest MAE on estimated A). Error obtained with EDec is on average 8% larger than the error obtained with RefFreeEwas and 2% larger than MeDeCom. We note that for the purpose of comparison we only performed Stage 1 of EDec and did not perform Stage 0, as recommended in the original EDec publication [4].

These results suggest that the differences between the tested algorithms are minor when default parameters are used and no filters are applied on the provided DNA methylation probes. The main variations in performance are related to simulation parameters, such as sample size (n) or inter-sample proportion variability (α_0_).

### Different strategies to initialize matrix factorization

Optimization algorithms implemented in MeDeCom, EDec and RefFreeEWAS start with an initial condition for either T, the cell type-specific methylation matrix, or A, the cell type proportion matrix.

**Fig. 2:**
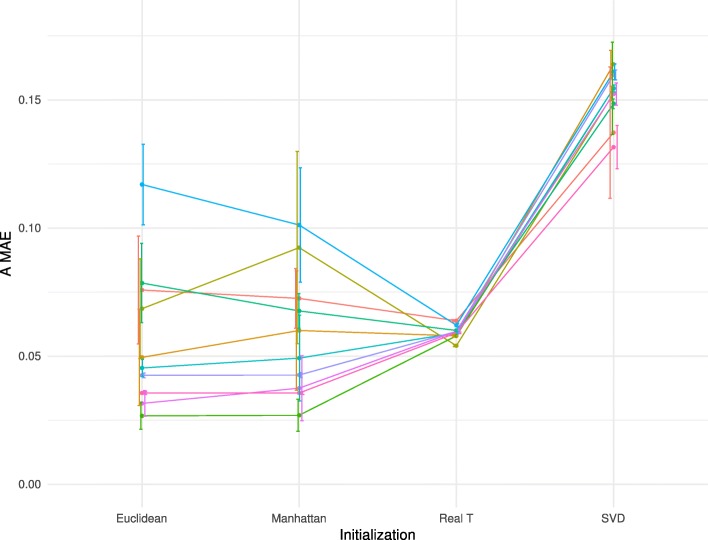
Impact of algorithm initialization of RefFreeEWAS method performance. `A MAE` is shown for 10 D matrices (mean value of 10 random noises applied on D) computed from 10 random A. Each color represents a different simulated A. Error bars represent standard deviation on 10 random noises. The following parameters were used to simulate D: K = 5, α_0_ = 1, *ε* = 0.2, G = 1 and *n* = 100). Euclidean corresponds to RefFreeEWAS::RefFreeCellMixInitialize function applied with the default parameter dist.method = “euclidean”. Manhattan corresponds to RefFreeEWAS::RefFreeCellMixInitialize function applied with the parameter dist.method = “manhattan”. Real T corresponds to RefFreeEWAS::RefFreeCellMix used with the parameter mu0 = real_T, with real_T the matrix composed of the 5 cell types used to simulate D. SVD corresponds to RefFreeEWAS::RefFreeCellMixInitializeBySVD function with default parameters

RefFreeEWAS initializes the T matrix. To explore the role of T initialization, we run the RefFreeEWAS package on 10 D matrices (generated from 10 random simulations of an A matrix with the following parameters: K = 5, *n* = 100, α_0_ = 1 and *ε* = 0.2). We use the following initialization schemes: K averaged methylation profiles are derived from the D matrix either by hierarchical clustering (estimation of the mean methylation of the K first clusters using a complete linkage method) based on (1) Euclidean or (2) Manhattan distances; either by (3) singular value decomposition (SVD) (corresponding to the K highest singular values) with discretized methylation values (0/1, 4) the ground truth corresponding to real T matrix used in the simulations is also tested (4) (Fig. 2, Additional file 2: Table S1, see Material and Methods for details). The RefFreeEWAS outcome varies significantly according to how it is initialized, especially when T is initialized by hierarchical clustering applied on the D matrix (estimation of the mean methylation of the K first clusters). Indeed, these two approaches based on hierarchical clustering display a high variability depending on the random simulations of A. Hierarchical clustering based on Euclidean distance or Manhattan distance performs similarly (error ranging from 0.022 to 0.135 for Euclidean distance, and from 0.022 to 0.138 for Manhattan distance). In some cases, they even outcompete initialization with the real T matrix used for simulations (0.059 mean MAE for real T), whereas the SVD approach perform systematically worse (0.152 mean MAE). Surprisingly, the effect of T initialization is highly dependent on the inter-sample variation in mixture proportion (α_0_). When variation of proportion is low (α_0_ = 10,000), SVD initialization is more efficient than hierarchical clustering (0.077 mean MAE for SVD-based initialization versus 0.23 mean MAE for clustering-based initialization) (Additional file 1: Figure S5, Additional file 2: Table S2).

MeDeCom performs multiple random initializations of A, the cell type proportion matrix, and EDec performs initialization with random guesses of proportions of cell types in each sample (randomized A generated from a Dirichlet distribution meeting boundary conditions of 0 < A < 1). To roughly estimate the differences between these two approaches on the outcome of the deconvolution algorithms, we run MeDeCom (without the regularization function: parameter lambda = 0) and EDec on 10 independent D matrices (generated from 10 random simulation of matrices A, *n* = 100, α_0_ = 1 and *ε* = 0.2) (Additional file 1: Figure S6). Interestingly, both approaches give similar results. There appears to be a range of errors across 10 different D matrices simulated for both methods tested, with 3 D matrices showing a high standard deviation across 10 random noises (Additional file 1: Figure S6), suggesting these initialization methods may show sensitivity as well in certain situations.

In summary, the strategies of initialization can have important impacts on method performance, some of them being highly dependent on the composition of the original D matrix. Further work is therefore needed to better understand the relationship between the initialization of the algorithm and method performance.

### Feature selection and accounting for confounders

Variation in DNA methylation is associated with different factors (such as age, sex, batch effects, etc.) that are not always related with cell-type composition. A popular assumption is that removing probes by feature selection will improve performance of deconvolution methods. Yet, such an approach may also discard relevant biological information [13].

**Fig. 3:**
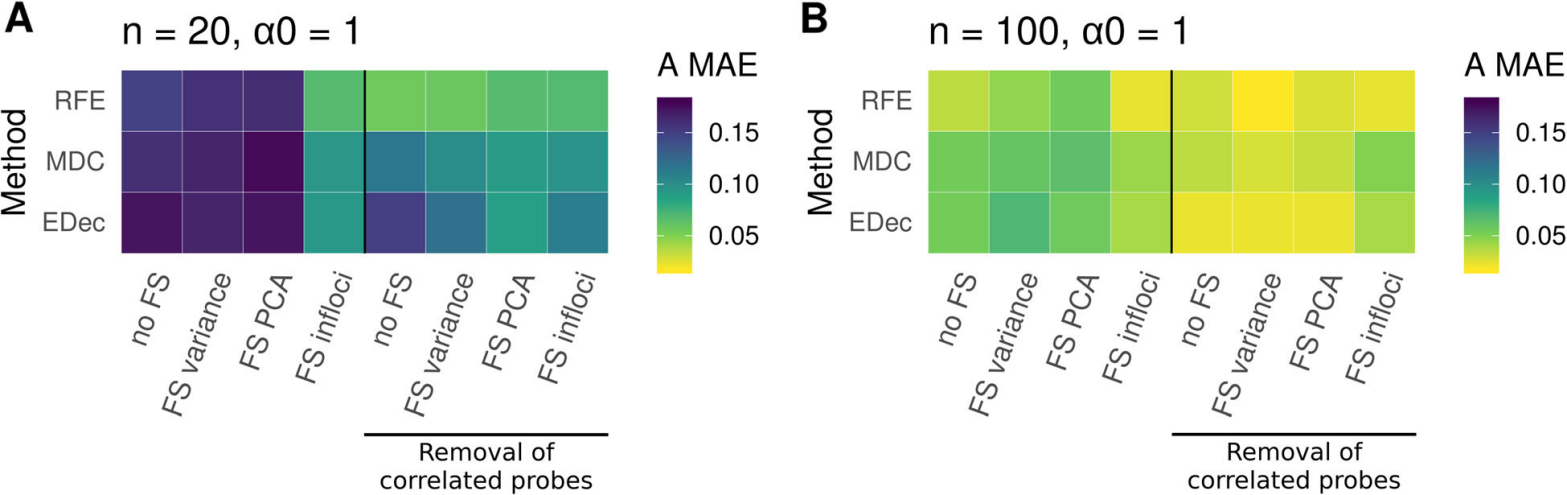
Impact of pre-processing on method performance. Heatmap of method performances (`A MAE`: Mean Absolute Error on estimated A, the matrix of cell proportions). RFE stands for RefFreeEWAS, MDC for MeDeCom and EDec for EDec stage 1. All algorithms are run on 10 D matrices: 10 different random noises *ε* were simulated on one matrix D computed from one simulated A matrix. In each heatmap, the left panel corresponds to algorithms run without accounting for confounders (no removal of confounding probes), the right panel corresponds to algorithms run accounting for confounders (removal of confounding probes by linear regression). In each case, different types of feature selection (FS) are tested: no FS = no feature selection, FS variance = selecting probes with high variance (var > 0.02), FS PCA = selecting probes highly correlated with the 4 first PCs (*p*-value < 0.1), FS infloci = selecting probes expected to biologically vary in methylation levels across constitutive cell types. **a** Simulations were performed with the following parameters K = 5, *n* = 20, α_0_ = 1, *ε* = 0.2 and G = 1. **b** Simulations were performed with the following parameters K = 5, *n* = 100, α_0_ = 1, *ε* = 0.2 and G = 1. The number of conserved probes is display Additional file 2: Table S1

Because probe selection always involves probe removal, we evaluate to what extent removing probes (i.e. Feature Selection, FS) impacts estimation error. In the previous section, all the 23,381 probes were used by the three non-negative matrix factorization algorithms. Here, we apply different types of feature selection and measure their impacts on deconvolution errors. First, we perform feature selection (FS) without removal of confounding probes (Fig. 3a and b, left panel). When keeping only the most variable probes, or the ones that are the most correlated with principal components (PCs), error remains similar to when using no FS (FS variance and FS PCA, resp. in Fig. 3). When keeping only cell-type variable informative probes based on literature curation and use of publicly available reference cell profiles as surrogates (FS infloci, as suggested in the EDec method, see Material and Methods for probe selection information), we find a large reduction of error (47% error reduction on average with 20 patients and 26% with 100 patients). Then, we remove confounding probes, which corresponds to ~ 1000–2000 probes significantly correlated with confounder variables (such as age, sex, etc.) with an adjusted false discovery rate (FDR) threshold of 15% [15].We subsequently observe a substantial reduction of error (33% in average with 20 patients and 35% with 100 patients). Other FDR thresholds between 5 and 20% provide similar error measures (Additional file 1: Figure S7). After removal of correlated probes, there is no systematic advantage in filtering additional probes by feature selection (see Fig. 3a and b, right panel). For each deconvolution software, best performances are always obtained, with both 20 and 100 patients, when removing probes correlated with confounders. However, the positive impact of removing confounding probes is only observed when inter-sample variation in mixture proportion is high (α_0_ = 1). When inter-sample variation is low (α_0_ = 10,000), the deconvolution is much more complicated, which could explain why we do not observe reproducible improvement of error detection after removing confounding probes in this case (Additional file 1: Figure S8).

In summary, we highly recommend to systematically remove possible confounding probes to account for confounders. Filtering based on biologically informative loci, after removing of confounding probes, can also be an interesting approach, if the investigated biological system is properly defined. In the rest of the paper, removal of confounding probes is always considered before using MeDeCom, and RefFreeEwas whereas EDec infers informative probes from any available references (in EDec Stage 0, as also illustrated in its vignette).

### Choice of the number of cell types K

We tested several methods to choose the number of cell types K including the scree plot based on PCA of the D matrix, a cross validation score provided by MeDeCom, a deviance statistic estimated with bootstrap provided by RefFreeEWAS, and contrast those to the “best fit” and stability methods used by EDec.

**Fig. 4:**
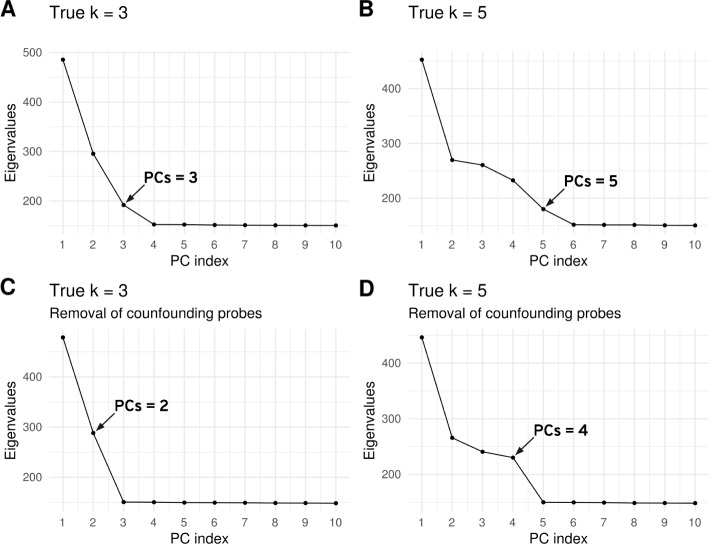
Determining K with PCA scree plot. To choose K, we recommend to use Cattell’s rule, calculating the estimated K as K = PCs + 1. The number of PCs chosen by the Cattel’s rule is shown with an arrow. The D matrix was simulated with the following parameters: *n* = 100, α_0_ = 1, *ε* = 0.2, G = 1 and K = 3 (**a** and **c**) and n = 100, α0 = 1, *ε* = 0.2, G = 1 and K = 5 (**b** and **d**). **a, b** Scree plot of PCA applied on D matrix before removal of confounding probes (23,381 probes). **c**, **d** Scree plot of PCA applied on D matrix after removal of confounding probes (22,551 probes in **C**, 22,532 probes in **d**)

First, we look at the scree plot by plotting the eigenvalues of the D matrix in descending order (Fig. 4). For choosing K, we use Cattell’s rule, which states that components corresponding to eigenvalues to the left of the straight line should be retained [16]. When the actual number of different cell types is equal to K, we expect that there are (K-1) eigenvalues would correspond to the mixture of cell types and that other eigenvalues would correspond to noise (or other unaccounted for confounders). Indeed, one PCA axis is needed to separate two types, two axes for three types, etc. However, when not accounting for confounders, Cattell’s rule overestimates the number of PCs and suggests to choose 3 and 5 PCs (i.e. K = 4 and K = 6) whereas the correct answers are K = 3 and K = 5 respectively (Fig. 4a, b). When accounting for confounders by removing probes significantly correlated with confounders, Cattell’s rule provides the correct answer of 3 and 5 cell types (2 or 4 PCs) (Fig. 4c, d). Finally, we observe that varying the FDR threshold used to select confounding probes has no impact of the choice of K using Cattell’s rule (for all thresholds tested, the estimation of K is correct, Additional file 1: Figure S9).

The cross-validation score provided by MeDeCom gives similar choices of K as the scree plot. When not accounting for confounders, graphical inspection of the decay of cross-validation error, as a function of the number of cell types, suggests to keep K = 4 or K = 6 cell types. When accounting for confounders, it suggests to keep K = 3 or K = 5 cell types, which are the correct answers, yet the distinction is less obvious for K = 5 (Additional file 1: Figure S10 right panel).

The bootstrap estimation of the deviance performed by RefFreeEWAS algorithm provides different answers from the previous two approaches. When the exact value of the number of cell types is K = 3, the minimum value of deviance is reached at the correct value of K = 3, whether confounders were accounted for or not. When the exact value of the number of cell types is K = 5, the deviance statistic indicates to choose a K = 4 or 5 whether confounders were accounted for or not (Additional file 1: Figure S10 left panel).

**Fig. 5:**
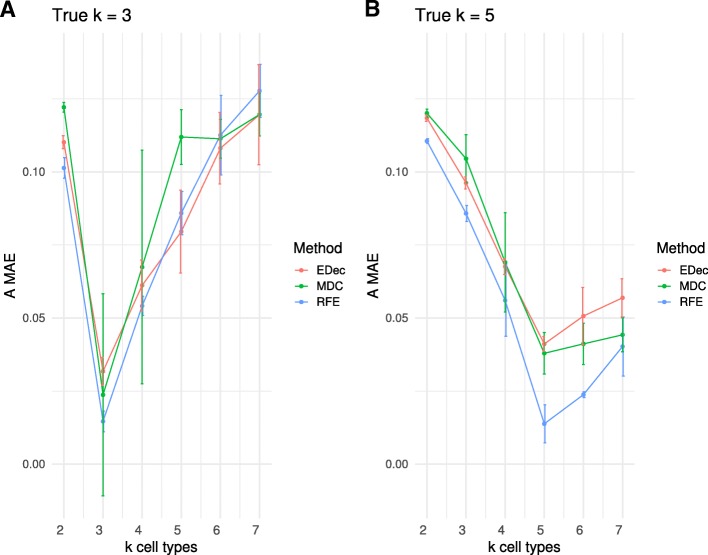
Impact of K selection on algorithm performance. `A MAE` is shown for D matrices (mean value of 10 random noises applied on D) computed from 1 random A. Each color represents a different method. Error bars represent standard deviation on 10 random noise realizations. RFE stands for RefFreeEWAS, MDC for MeDeCom and EDec for EDec stage 1, each method was applied with various imposed K parameters (from 2 to 7). **a** The following parameters were used to simulate D: K = 3, α_0_ = 1, *ε* = 0.2, G = 1 and *n* = 100. RFE and MDC methods were run after removal of confounding probes (between 22,517 and 22,624 remaining probes), EDec was run on informative loci, as recommended by the method’s authors (614 remaining probes). **b** The following parameters were used to simulate D: K = 5, α_0_ = 1, *ε* = 0.2, G = 1 and n = 100. RFE and MDC methods were run after removal of confounding probes (between 22,551 and 22,602 remaining probes), EDec was run on informative loci, as recommended by the method’s authors (614 remaining probes)

Lastly, we investigate to what extent inference of cell type proportions is robust with respect to the choice of K. We find that the choice of K has a strong influence on inference of cell type proportions. Underestimation of K has a large impact on measured error, and overestimation of K, albeit to a lesser extent, also increases measured error (Fig. 5). For instance, when the correct K is equal to 3, using K = 4 instead of K = 3 provides an error measure that is at least twice as large regardless of the deconvolution method that is used. Overestimation of K leads to large increase of error, even if our MAE-computing algorithm only retains the 3 cell types that minimize MAE error among the 4 inferred cell types.

These results suggest that the choice of K can be reliably guided by a scree plot based on PCA, after accounting for confounders.

Alternatively, the best performance at true K-s in Fig. 5 suggests a different approach where K is selected after running deconvolution for various values of K and choosing the K with best performance. EDec adopts this general idea by selecting the K that explains the most variance (achieves the best fit of D) and the largest K that shows stable estimates of both the A and T matrices. The stability is tested by taking at least 3 subsets (consisting of randomly selected 80% of the sample profiles) and measuring the similarity of estimates for A and for T across the 3 subsets.

### Biological interpretation of recovered methylation profiles T

We next compare the estimated matrix of average cell type-specific methylation profiles (matrix T) with the real methylation profiles of cell types used to simulate the datasets.

**Fig. 6:**
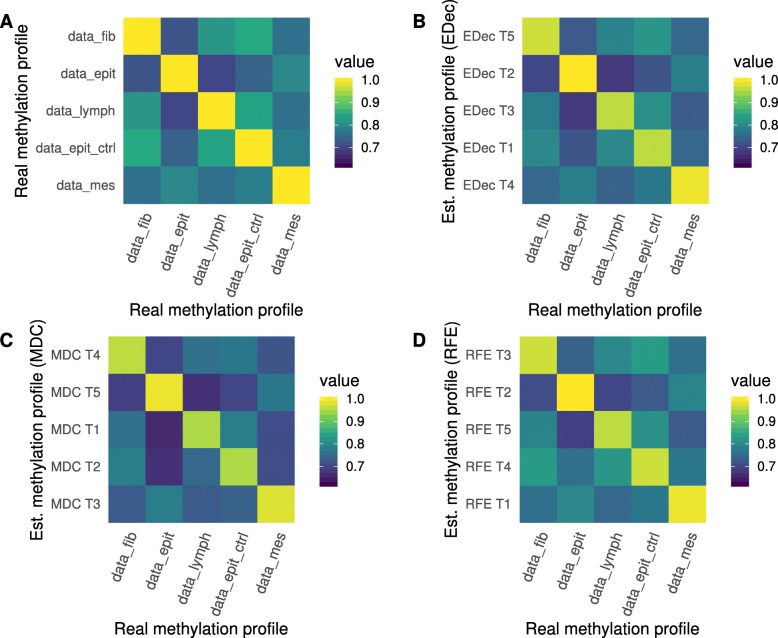
Correlation between estimated and real cell type-specific methylation profiles. Heatmap of the correlations between cell type-specific methylation profile used for the simulation and cell type-specific methylation profiles estimated (Est.) by different methods. In (**a**), the correlation between different cell types used for the simulation of the T matrix (data_fib = fibroblast, data_epith = cancerous epithelial, data_lymph = T lymphocytes, data_epit_ctrl = healthy epithelial and data_mes = cancerous mesenchymal). We applied EDec (**b**), MeDeCom (**c**) and RefFreeEwas (**d**) on a representative simulation of 100 patients (α_0_ = 1, *ε* = 0.2, G = 1, K = 5) after the removal of confounding probes by linear regression (22,483 remaining probes). We used the Pearson method to compute the correlation between the estimated cell type-specific methylation profiles and real cell type-specific methylation profiles used for the simulation

To assess the similarity of reconstructed methylation profiles, we run the three algorithms on a representative D matrix, with *n* = 100 patients. We then draw a heatmap representing the level of correlation between estimated methylation profiles and reference methylation profiles. When the inter-sample variation is high (α_0_ = 1), we observe that all the deconvolution methods tested succeed to properly estimate the cell type-specific methylation profiles and to robustly identify corresponding reference cell types (Fig. 6). When the inter-sample variation is low (α_0_ = 10,000), all methods fail to identify reference cell types (Additional file 1: Figure S11), which is consistent with the results observed in Additional file 1: Figure S8. Importantly, in a real setting, true methylation reference profiles are not available, and the identity of the cell-types present is unknown, which strongly complexifies the biological interpretation and the annotation of the obtained cell types.

Altogether, this indicates that reducing the inter-sample variability will impact both identification of cell type proportion and the identification of existing cell type-specific methylation profiles.

### Recommendation and application of guidelines on simulated and real datasets

We have conducted extensive benchmarking of the core deconvolution step shared by three algorithms dedicated to reference-free cell-type heterogeneity quantification from DNA methylation datasets. We identify the following critical steps influencing method performance: i) accounting for confounders, ii) feature selection and iii) the choice of the number of estimated cell types. To account for confounders, we suggest to remove probes associated with the measured confounders. We suggest to use a large FDR threshold, preferring to remove too many probes rather than keeping probes influenced by confounders, given the large (> 20,000) initial number of probes. When probes associated with confounders are not removed, the number of cell types is overestimated, capturing the additional dimension of confounders (Fig. 4). We recommend that care to be taken to choose the right number K of cell types, as the outcome of deconvolution may strongly depend on K. One such method is the scree plot and Cattell’s rule, which states that eigenvalues corresponding to true signal are to the left of the straight line (Fig. 4). The number of cell types to consider is equal to the number of eigenvalues to keep plus one. Choice based on Cattell’s rule is robust with respect to the FDR threshold used when discarding probes associated with confounders (Additional file 1: Figure S9). An alternative, implemented by EDec, is to select the largest K that shows stable estimates of both the A and T matrices. Lastly, we suggest to use feature selection and to further reduce the number of probes by focusing on probes that are informative of cell types (as previously implemented in Stage 0 of EDec) or the highest variance markers if no biological information is available. Further reducing the number of probes marginally affects estimation error (Fig. 3), but can substantially reduce computational running time (Additional file 2: Table S3).

**Fig. 7:**
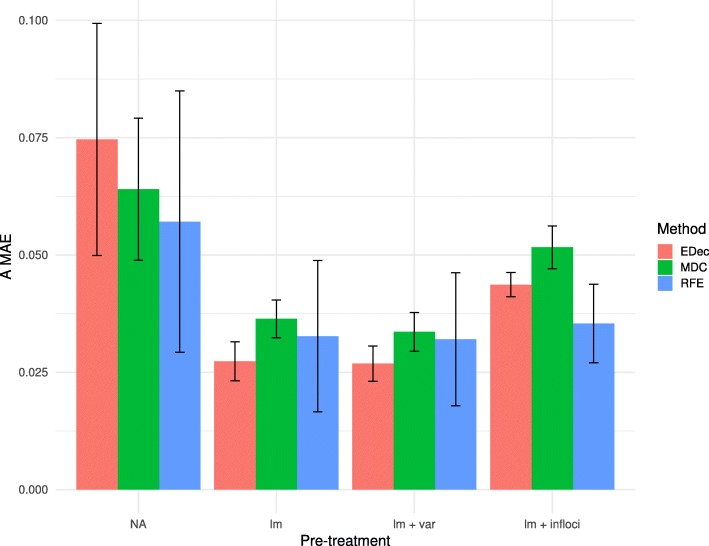
Comprehensive comparison of the pre-processing pipeline. Histogram of `A MAE` (`A MAE`: Mean Absolute Error on estimated A, the matrix of cell proportions) for 10 D matrices (mean value of 10 random noises applied on D) computed from 10 random A. Each color represents a different method. Error bars represent standard deviation on 10 random noises. RFE stands for RefFreeEWAS, MDC for MeDeCom and EDec for EDec stage 1. The following parameters were used to simulate D: K = 5, α_0_ = 1, *ε* = 0.2, G = 1. The methods were run without pre-processing (NA), after removal of confounding probes by linear regression (lm), after removal of confounding probes and filtering for the most variable probes (lm + var), and after removal of confounding probes and filtering of probes expected to biologically vary in methylation levels across constitutive cell types (lm + infloci)

When applied on simulated datasets, once the markers and the number of cell types have been chosen, the core deconvolution step implemented by one of the three packages would provide comparable estimates sample-specific cell-type proportions (Fig. 7). In order to test the performances of our pipeline on real heterogeneous tumor samples, we applied it to TCGA lung adenocarcinoma cohort (LUAD) and TCGA lung squamous cell carcinoma cohort (LUSC) [17, 18]. We compared the consistency of immune cell (IC) fraction estimated by our pipeline with the IC fraction estimated by the reference-based EpiDISH algorithm [19] and the IC fraction estimated by ESTIMATE algorithm [20] on RNA-seq profiles (Additional file 1: Figure S12 and S13). For both lung cancers, we observed consistency between our reference-free pipeline estimates and independent estimates (average correlation with reference-based estimate of 0.73 for LUAD and 0.78 for LUSC, and averaged correlation with RNA-seq based estimate of 0.66 for LUAD and 0.75 for LUSC). These data demonstrate that our pipeline can achieve good performances on real heterogeneous samples.

## Conclusion

**Fig. 8:**
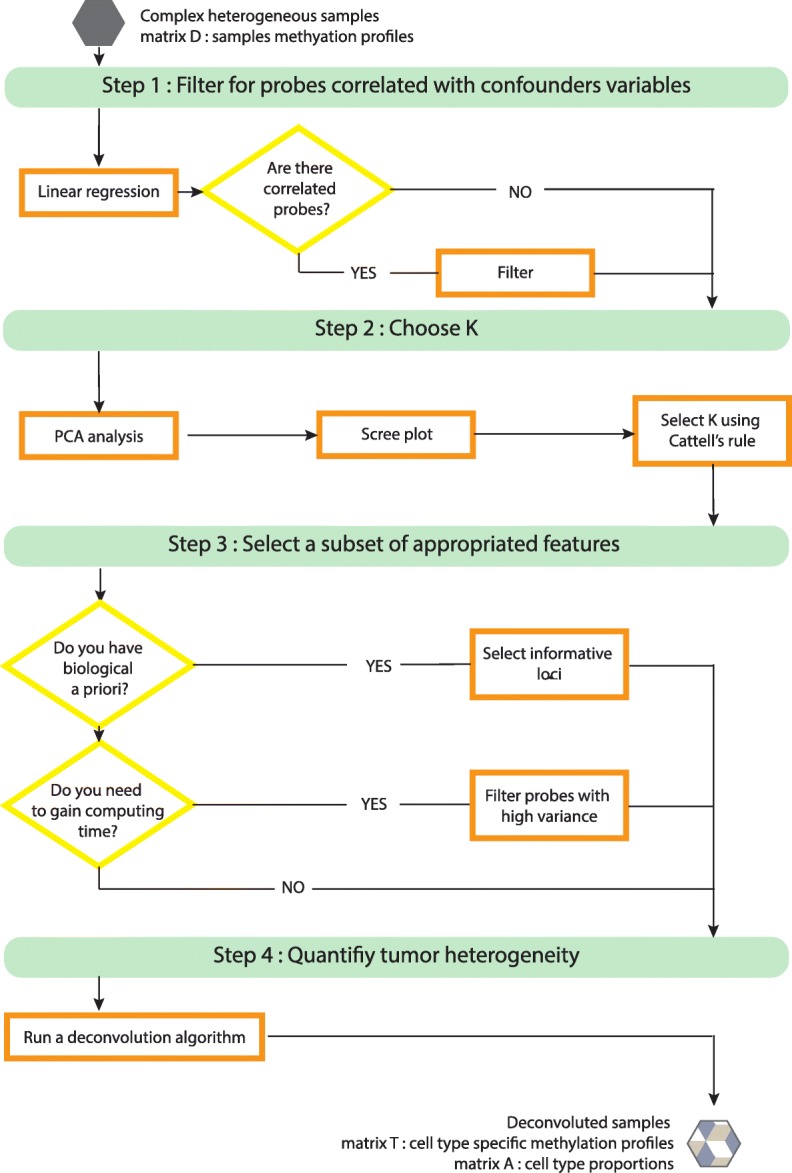
Recommendations and benchmarking pipeline

Based on lessons learned from the simulation experiments, we developed a benchmark pipeline to estimate cell-type proportions that addresses the presence of confounders and other key factors affecting performance of deconvolution algorithms (Fig. 8). We anticipate that this benchmark pipeline will help catalyze wide adoption of deconvolution methods and accelerate improvement of deconvolution pipelines by (1) helping validate other deconvolution pipelines by demonstrating concordant results; (2) serving as a benchmark for demonstrating improved performance of other pipelines; (3) providing a starting point (“toolkit”) for development of new pipelines.

We note that the benchmark pipeline is not experimentally validated nor it is systematically compared as a whole against more complex pipelines that include expression data (e.g., all stages of EDec pipeline). In our experience, no deconvolution pipeline can be expected to provide accurate solutions when applied “out of the box” to a new tumor type. Tuning and validation are required in the context of each tumor type, using resources and information that may be tumor-type specific. In that sense, deconvolution may be thought of as a computational modeling approach that goes hand-in-hand with experimentation.

## Discussion

### Initialization

The software MeDeCom, EDec and RefFreeEwas have different methods to initialize the deconvolution algorithm. MeDeCom performs multiple random initialization of the matrix of proportion A. EDec performs initialization with random draws of proportions of cell types in each sample. RefFreeEwas initializes the matrix of cell types using reduction dimension or clustering techniques depending on a user-defined option. For RefFreeEwas, we find that clustering techniques provide the best option in the favorable scenario when cell-type proportions strongly differ between individuals (Fig. 2). However, in the less favorable scenario where cell-type proportions are more similar between individuals, initialization based on singular value decomposition should be preferred (Additional file 1: Figure S5). Differences of performance according to initialization are substantial and depending on initialization, error measures can vary by a factor of two. The fact that deconvolution methods depend on initialization indicates that there is room for improvement either by finding an optimal initialization strategy or by using an ensemble method that combines or averages several deconvolution solutions [21, 22].

### Evaluation of performance

To estimate performances of methods, we used the MAE metric. A similar alternative metric, the root mean square error (RMSE) gave equivalent performance evaluation (Additional file 1: Figure S14). Internal steps of the MeDeCom algorithm use a leaving-columns-out Cross-Validation Error (CVE) to choose regularization parameter λ and number of cell types K. In our study, we consider a grid of six values for the regularization parameter λ (0, 0.00001, 0.0001, 0.001, 0.01, 0.1). Interestingly, the optimum lambda selected by the CVE approach does not always perform better than λ = 0, when evaluated by the MAE metric (Additional file 1: Figure S15). Although it is difficult to assess the biological relevance of each possible error metric, we would like to emphasize that the choice of evaluation metrics is an important parameter when conducting a benchmarking study. In addition, evaluation based on simulations remain limited compared to evaluation based on real tumor datasets, because the in silico simulation does not model all the biological properties of the system, such as changes in methylation due to cell-cell interaction. However, we are still lacking real tumor dataset with accurate quantification of tumor heterogeneity. Deconvolution algorithms evaluation will then be significantly improved with the generation of dedicated in vivo benchmarking dataset

### Data challenge, collaborative and open science

Our work strongly benefits from a data challenge format where different pipelines were proposed and evaluated. We gathered methylation deconvolution experts for a week of brainstorming on this benchmarking issue. We used the resulting ideas and computational methods to construct several pipelines which we evaluated in the paper. Thus, all challenge participants are referred as consortium authors of the paper. As a key deliverable of the project, to facilitate wide application of reference-free deconvolution and also pipeline development by the community, we develop a benchmark pipeline and release it as an R package (Fig. 8 and R package *medepir*).

## Material and methods

### Matrix factorization

We assume D is a (M × N) methylation matrix composed of methylation value for N samples, at M CpG methylation sites. Each sample is constituted of K cell types. We assume the following model: D = TA, with T an unknown (M × K) matrix of K cell type-specific methylation reference profiles (composed of M sites), and A an unknown (K × N) proportion matrix composed of K cell type proportions for each sample. In the methods tested here, A and T are found using matrix factorization, which consists of minimizing the error term ||*D* − *TA*||_2_, with constraints on methylation values, 0 ≤ *A* ≤ 1 and 0 ≤ *T* ≤ 1, and on proportions ${\sum}_{k=1}^K{A}_{kn}=1$ (MeDeCom and EDec) or ${\sum}_{k=1}^K{A}_{kn}\le 1$ (RefFreeEWAS), where *A*_*kn*_ is the proportion of the n*th* sample for the k*th* cell type*.* MeDeCom uses an additional regularization function, which depends on a regularization term, which is weighted by a hyperparameter λ, that favors methylation values close to 0 or 1.

$$ \lambda {\sum}_{k=1}^K{\sum}_{n=1}^N\omega \left({T}_{kn}\right)\kern0.5em \mathrm{with}\kern0.5em \upomega (x)=x\left(1-x\right). $$

### TCGA DNA methylation data

We used the The Cancer Genome Atlas (TCGA) lung cancer dataset (LUAD and LUSC) as an example biological dataset. We downloaded and processed level 3 Illumina 450 k and 27 k methylation data (beta values) from TCGA, with associated metadata. To extract confounding factors parameters, we used clinical data associated with normal (non-tumor) samples (LUAD, *n* = 56 and LUSC, *n* = 69). When applying *medepir* pipeline on real heterogeneous dataset, we selected 456 tumor samples of DNAm 450 K for LUAD and 370 tumor samples of DNAm 450 K for LUSC. Difference between type I and type II probes was normalized with the function wateRmelon::BMIQ [23] using [24] as reference for the probe types.

### Datasets used for simulations

We simulated synthetic DNA methylation mixtures from cell lines and primary cells (27 K or 450 K DNA methylation, see Additional file 2: Table S4). We used a variety of cell type-specific methylation profiles to simulate lung cancer heterogeneity, including cancerous and normal epithelial cells, cancerous mesenchymal cells, normal fibroblasts for stromal cells, and T lymphocytes for immune cells. We selected M = 23,381 probes using the following criteria: (i) intersect between the Illumina Infinium 27 k and Illumina Infinium 450 k DNA methylation array, and (ii) non-null probes in 100% of LUAD and LUSC methylation datasets. We tested whether Illumina 450 k type I and type II probes have an effect on algorithms’ performances using the BMIQ packages that adjusts type II design probes distribution. Algorithms performances were similar on simulations using 450 k data after or before BMIQ correction (Additional file 1: Figure S16), suggesting type I or type II probes design of Illumina 450 k has no significant effect on our specific simulation design.

### Simulation models

Simulated D matrix were obtained using D = TA model

#### Simulation of the A matrix

The cell type proportion A matrix is simulated by a Dirichlet distribution with parameters defined for 10% of fibroblast, 60% of cancerous epithelial, 5% of T lymphocytes, 15% of control epithelial and 10% of cancerous mesenchyme. The mixture proportions are sampled from a Dirichlet distribution with parameters generating sets of proportions more or less variable across the sample population. The parameter *α* 0, which defines the variability across the sample population, is set to 1 by default. For simulation with only three cell types, we used 20% of fibroblast, 70% of cancerous epithelial and 10% of T lymphocytes.

#### Simulation of the T matrix

To initiate T, we use the purified cell-types methylome describes in Additional file 2: Table S4. Once the initial T matrix is defined, we apply a series of confounding effects using parameter extracted from the clinical data associated with LUAD and LUSC cohorts’ normal samples.

First, we generate an individual-specific T matrix, accounting for two major biological confounders for methylation, which are sex and age.

To identify effects of sex, we performed linear regression of methylation by sex in the TCGA dataset and detected 1397 probes correlated with sex (*p*-value < 0.01). Given that the majority of cell lines used to construct the initial T matrix were derived from male individuals, we used the corresponding linear regression coefficients to shift accordingly methylation value of female-associated T matrices. We used the same sex coefficient for each cell type represented in the T matrix.

To identify effects of age, we performed linear regression of methylation by age in the TCGA dataset and identified 113 probes correlated with age (*p*-value < 0.05). We used this linear model to generate an individual-specific methylation profile, according to its age. Then, we arbitrarily decided to assign these 113 methylation values to the normal epithelial cell type. For each probe associated with age, we then applied a normalization coefficient (ratio of each cell type to the epithelial cell type computed in the initial T matrix) to modify methylation values of the remaining cell types. Our simulation scheme implicitly assumes that age has the same effect whatever the cell type.

#### Simulation of the D matrix

Second, we decided to account for technical confounders. We calculated 22 median plate-effects (TCGA experimental batch effect) using 1000 random probes. For each probe, we modeled plate effects using multiplicative coefficients that measure the ratio of mean methylation values of a plate on mean methylation of the (arbitrarily) 1st plate. Each coefficient is estimated by the median of the 1000 ratios of methylation values. These multiplicative coefficients are then used on all probes to model batch effects on the matrix D of individual convoluted methylation profile.

Finally, we added Gaussian noise on the matrix of convoluted methylation profiles D. By default, we used the Gaussian parameters mean = 0 and sd = 0.2. In case noise generated methylation values larger than 1 or smaller than 0, noise was not added to the methylation value.

The simulation function is accessible using our R package *medepir*.

### Software usage

We follow publication guidelines and default parameters for each method (see Material and Methods for details in software usage). RefFreeEwas was used with the function “RefFreeCellMix” with 9 iterations and remaining parameters set to default, unless specified otherwise. MeDeCom was used with the function runMeDeCom with the following parameters: NINIT = 10, NFOLDS = 10, ITERMAX = 300, lambdas = c(0, 0.00001, 0.0001, 0.001, 0.01, 0.1), if unless specified otherwise. EDec was used with the function run_edec_stage_1 with default parameters, unless specified otherwise. When testing the initialization of matrix T in RefFreeEWAS algorithm, we used the following functions: RefFreeEWAS::RefFreeCellMixInitializeBySVD(D, type = 1), RefFreeEWAS:: RefFreeCellMixInitialize(method = “ward”, dist.method = “euclidean”), RefFreeEWAS:: RefFreeCellMixInitialize(method = “ward”, dist.method = “manhattan”). Each RefFreeEWAS initialization method estimates an averaged methylation profile for K components from the initial D matrix (either by hierarchical clustering on D, or by singular value decomposition of D). The SVD method initializes the reference-free cell mixture deconvolution using an ad-hoc method attempting to obtain T by discretizing to 0/1 the U matrix (left-singular vectors obtained by SVD of D). The threshold used for binarization is the median value of each row of the U matrix.

### Confounding factor detection

Accounting for confounding probes was performed using linear regression for each confounding factor (based on clinical metadata extracted from TCGA LUAD and LUSC cohorts). We control for FDR using Benjamini-Hochberg correction. For each confounder variable, we removed probes that are significantly associated with the confounder variable using an FDR threshold of 0.15.

### Feature selection

To enhance the precision of the method, we can select the more informative probes. For this, we tested three methods: (i) selecting probes with high variance (var > 0.02), (ii) selecting probes highly correlated with the first four PCs (*p*-value < 0.1) [25, 26], and (iii) selecting probes expected to biologically vary in methylation levels across constitutive cell types, as described in EDec method (see below for detailed explanation), the corresponding probes are depicted in Additional file 2: Table S5.

Probes on the HM450 array which were previously shown to be cross reactive, sex-specific, contain missing values, or contain SNPs were filtered using http://zwdzwd.github.io/InfiniumAnnotation#current. Additionally, probes with non-zero covariate effects as determined by sparse regression (R package lfmm, function lfmm::lfmm_lasso) with a subset of covariates (t, n, m, age, dead, center, expo and sex) were filtered. Publicly available 450 K and 27 K cell reference profiles were downloaded from GEO (https://www.ncbi.nlm.nih.gov/geo/) and grouped into 5 representative cell types predicted to constitute the bulk tissue: stroma (5 cancer-associated fibroblast and 5 fibroblast profiles), cancer (21 lung cancer cell profiles), epithelial (7 lung epithelial references), endothelial (9 references), and immune (19 references consisting of a mixture of T-reg, monocyte, granulocyte, neutrophil, CD4+ T-cell, and CD + 8 T-cell profiles). Using these 5 reference groups, informative loci were chosen as previously described [4]. Briefly, 500 informative loci were chosen using EDec’s stage 0 command using the “one.vs.rest” option with a *p*-value of 1e-4. 100 informative loci were then added to this set of 500 using the stage 0 command with the “each.pair” option for each pairwise comparison between the epithelial and stroma groups and the epithelial and immune groups to yield a final set of 614 unique informative probes (unique(500 + 100 epithelial vs. stroma + 100 epithelial vs. immune)).

### Performance evaluation

We evaluate algorithm performances by (i) computing the mean absolute error (MAE) on estimated A (the matrix of cell type proportion, of size KxN, with K the putative number of cell types, and N the total number of samples), defined as $MAE=\left({\sum}_{n=1}^N{\sum}_{k=1}^K\left|{Aest}_{nk}-{Areal}_{nk}\right|\right)/(NK),$ or (ii) computing the root-mean-square error (RMSE) on estimated A, defined as $RMSE=\sqrt{\ \left({\sum}_{n=1}^N\ {\sum}_{k=1}^K{\left({Aest}_{nk}-{Areal}_{nk}\right)}^2\right)/ NK\ }$, with n the total number of observations.

### Application to LUAD and LUSC cell-type heterogeneity deconvolution

*medepir* pipeline was applied on 456 samples of DNAm 450 K TCGA LUAD and 370 samples of DNAm 450 K TCGA LUSC. First, we removed all probes with NA in at least one sample or a beta-value of 0 in all samples. Then, we filtered probes using confounding factor detection (98,580 probes were removed in LUAD cohort, and 18,826 in LUSC cohort). With used PCA to estimate the number of cell types present in the mixtures (K = 5 in LUAD, k = 4 in LUSC) and we applied feature selection based on probes maximum variance. At the end, we kept 29,053 probes in LUAD cohort and 97,600 probes in LUSC cohort). EpiDISH algorithm was applied on 456 samples of DNAm 450 K TCGA LUAD and 370 samples of DNAm 450 K TCGA LUSC using the function “epidish” (by default parameters), with centEpiFibIC.m as reference. The proportion of immune cells (IC) was download on Estimate website (https://bioinformatics.mdanderson.org/estimate/disease.html) based on the RNA-seqV2 analysis of TCGA datasets. 449 samples of LUAD and 365 samples of LUSC were common between DNAm 450 K and RNAseq.

## Supplementary information

**Additional file 1: Figure S1.** Overview of in silico simulations. **Figure S2.** Variation of the estimated A MAE depending of the number of patients. **Figure S3.** Distribution of cell types proportions according to the parameter α_0_. **Figure S4.** Variation of the estimated A MAE depending of the cells used for simulations. **Figure S5.** Impact of the initialization method of RefFreeEwas for a stable Dirichlet simulation. **Figure S6.** Effect of A matrix initialization on algorithms performances. **Figure S7.** Impact of the FDR threshold for the removing of confounding factors. **Figure S8.** Impact of pre-processing for a stable Dirichlet. **Figure S9.** Determining K is robust to variations in accounting for confounders. **Figure S10.** Determining K by RefFreeEWAS and MeDeCom. **Figure S11.** Correlation between estimated and real cell type-specific methylation profiles for a Dirichlet of α_0_ = 10,000. **Figure S12.** Efficiency of the pipeline on heterogeneous clinical LUAD tumor samples**. Figure S13.** Efficiency of the pipeline on heterogeneous clinical LUSC tumor samples. **Figure S14.** Variation of the error metric: Mean Absolute Error and Root-Mean-Square Error. **Figure S15.** Impact of lambda parameter for MeDeCom. **Figure S16:** Effect of probe type on simulations

**Additional file 2: Table S1.** Number of remaining probes in Fig. 3. **Table S2.** Number of remaining probes in Additional file 1: Figure S8. **Table S3.** Mean execution time in Fig. 7 (minutes). **Table S4.** Table of cells used for simulations. **Table S5.** Informative loci.

## Data Availability

The *medepir* R package (DNA MEthylation DEconvolution PIpeline in R) can be downloaded at https://github.com/bcm-uga/medepir. Documentation and usage examples are also available on the same page. All the datasets associated with this publication (lung cancer patient metadata) can be found in the TCGA webpage.

## Abbreviations

CVE: Cross-validation error
DNAm: DNA methylation
EWAS: Epigenome wide association studies
FDR: False discovery rate
FS: Feature selection
IC: Immune cells
lm: linear model
LUAD: Lung adenocarcinoma
LUSC: Lung squamous cell carcinoma
MAE: Mean absolute error
MDC: MeDeCom
PCA: Principal component analysis
PCs: Principal components
RFE: RefFreeEWAS
RMSE: Root mean square error
sd: standard deviation
SNP: Single-nucleotide polymorphism
SVD: Singular value decomposition
TCGA: The Cancer Genome Atlas
var: variance
