# Supplementary material for: Guidelines for cell-type heterogeneity quantification based on a comparative analysis of reference-free DNA methylation deconvolution software
Source: BMC Bioinformatics. 2020 Jan 13;21:16. doi: 10.1186/s12859-019-3307-2 (PMC6958785; doi:10.1186/s12859-019-3307-2)
Supplement: Supplementary file 2 — Additional file 2: Table S1. Number of remaining probes in Fig. 3. Table S2. Number of remaining probes in Additional file 1: Figure S8. Table S3. Mean execution time in Fig. 7 (minutes). Table S4. Table of cells used for simulations. Table S5. Informative loci. [file 12859_2019_3307_MOESM2_ESM.docx]

#### Supplementary Table 1: Number of remaining probes in Figure 3

|  | **No FS** | **FS variance > 0.02** | **FS pval PCA < 0.1** | **infloci** |
| --- | --- | --- | --- | --- |
| **n = 20** | 23.381 | 9.875 | 5.957 | 614 |
| **n = 20 + removing of confounding probes** | 21.487 | 9.153 | 5.621 | 591 |
| **n = 100** | 23.381 | 9.593 | 9.017 | 614 |
| **n = 100 + removing of confounding probes** | 22.483 | 8.959 | 8.634 | 607 |

####

#### Supplementary Table 2: Number of remaining probes in figure S8

|  | **No FS** | **FS variance > 0.02** | **FS pval PCA < 0.1** | **infloci** |
| --- | --- | --- | --- | --- |
| **n = 20** | 23.381 | 9.082 | 3.953 | 614 |
| **n = 20 + removing of confounding probes** | 21.549 | 8.508 | 3.477 | 597 |
| **n = 100** | 23.381 | 7.742 | 4.271 | 614 |
| **n = 100 + removing of confounding probes** | 22.376 | 7.132 | 3.717 | 605 |

#### Supplementary Table 3: Mean execution time in figure 7 (minutes)

|  | **No pre-treatment** | **Removing of confounding probes** | **Removing of confounding probes + FS variance > 0.02** | **Removing of confounding probes + FS infloci** |
| --- | --- | --- | --- | --- |
| **Edec** | 5.602 | 24.905 | 9.232 | 0.295 |
| **MeDeCom** | 26.848 | 25.549 | 10.227 | 0.765 |
| **RefFreeEwas** | 0.285 | 0.273 | 0.104 | 0.009 |

####

#### Supplementary Table 4: Table of cells used for simulations

|  | **Cancerous epithelial** | **Cancerous Mesenchymal** | **Fibroblaste** | **T cell** | **Control epithelial** |
| --- | --- | --- | --- | --- | --- |
| **G1** | Cell line GSM1560930, 27k | Cell line GSM1560925, 27k | Cell line GSM1354676, 27k | Primary cells GSM1641099, 450k | Cell line GSM2743808, 450k |
| **G1bis** | Cell line GSM1560930, 27k | Cell line GSM1560925, 27k | Cell line GSM1354676, 27k | Primary cells GSM1641099, 450k + BIMQ | Cell line GSM2743808, 450k + BMIQ |
| **G2** | Cell line GSM1560911, 27k | Cell line GSM1560931, 27k | Cell line GSM1354675, 27k | Primary cells GSM1641101, 450k | Cell line GSM2743807, 450k |
| **G3** | Cell line GSM1560930, 27k | Cell line GSM1560925, 27k | Cell line GSM1354676, 27k | Primary cells GSM1641099, 450k | Primary cells GSM999346, 450k |
| **G4** | Cell line GSM1560930, 27k | Cell line GSM1560925, 27k | Cell line GSM1354676, 27k | Primary cells GSM1641099, 450k | Primary cells GSM999358, 450k |
| **G5** | Cell line GSM1560930, 27k | Cell line GSM1560925, 27k | Cell line GSM1354676, 27k | Primary cells GSM688851, 27k | Primary cells GSM1337281, 27k |

#### Supplementary Table 5: Informative loci

cg18509435

cg03608577

cg20542190

cg12242338

cg07349094

cg18963171

cg19680672

cg16869108

cg05788638

cg10917619

cg13445249

cg03602500

cg02311163

cg03872376

cg15374234

cg18841952

cg07950803

cg24355048

cg24423088

cg00463848

cg19486673

cg13897627

cg26135325

cg26530341

cg00041575

cg12417466

cg07374637

cg05615150

cg06183267

cg14366598

cg14153740

cg25890048

cg00895324

cg25119415

cg17657618

cg24338843

cg01637734

cg23338195

cg20856834

cg07525077

cg27431150

cg24272907

cg03914397

cg04574507

cg13765961

cg07548313

cg16812893

cg05767404

cg22325572

cg26799474

cg12910797

cg03547924

cg21949305

cg15720535

cg21453309

cg15475323

cg03171924

cg06469542

cg25509184

cg09061733

cg10942056

cg06490988

cg21129531

cg20308817

cg26200585

cg07197059

cg10574499

cg13705284

cg24019564

cg04527918

cg27635271

cg09440340

cg25431974

cg22764925

cg17791651

cg10266490

cg13641903

cg10257049

cg15901783

cg15083233

cg15149645

cg17465304

cg05724065

cg26521404

cg22396755

cg10282491

cg05606799

cg05590982

cg10046892

cg26884581

cg02324920

cg08044694

cg02838492

cg11052143

cg10280342

cg12782180

cg08519905

cg21488617

cg24989962

cg17349199

cg26606064

cg14859417

cg12513481

cg23606023

cg14371590

cg02490034

cg20373326

cg19138960

cg09037813

cg01222684

cg08314660

cg07498879

cg22972055

cg25313204

cg20622019

cg06465194

cg23797100

cg18345635

cg12041387

cg00661485

cg14754581

cg08077673

cg19539004

cg21614638

cg23663476

cg19863740

cg25947945

cg18997129

cg27433088

cg00503840

cg25949363

cg05670596

cg27016494

cg16363586

cg20583073

cg00499822

cg19248557

cg24861272

cg20080624

cg05656364

cg20876010

cg09954385

cg04947157

cg15164103

cg14236389

cg00412772

cg07816074

cg07665060

cg14417329

cg07359545

cg03386869

cg24541550

cg24619694

cg20356482

cg08615333

cg13053396

cg06630567

cg14137939

cg14918082

cg22979433

cg06589885

cg02506908

cg06290096

cg24365867

cg14027234

cg02586730

cg06339706

cg26215428

cg14297029

cg15679095

cg27256309

cg24315815

cg04106785

cg25182621

cg10857774

cg13521229

cg11213150

cg07260017

cg10222534

cg12069042

cg19466563

cg03389133

cg19664945

cg10635061

cg19433435

cg05955301

cg18003231

cg11042320

cg10362475

cg26924825

cg07168556

cg26220985

cg24447890

cg22416721

cg04999691

cg20368904

cg26453588

cg03870261

cg24340657

cg07354440

cg13030582

cg23130254

cg21577049

cg23092823

cg21815667

cg04797323

cg10756887

cg19352038

cg25465406

cg21790626

cg13035743

cg19797376

cg26620157

cg21233722

cg26189983

cg19358493

cg06825142

cg00347904

cg22709192

cg07696033

cg07634191

cg13577076

cg11108890

cg26069745

cg22341310

cg07533148

cg23587449

cg27188703

cg27009703

cg26113512

cg03874199

cg21238818

cg12265829

cg24516901

cg21529533

cg14056644

cg16761581

cg20616414

cg22377389

cg09516965

cg15540820

cg01335367

cg16158681

cg18536148

cg21604042

cg26416466

cg01683883

cg05345286

cg23887396

cg00363813

cg03421300

cg00744433

cg04245402

cg08687163

cg21459867

cg25612480

cg22477971

cg03109316

cg05445326

cg21484834

cg07785936

cg01731341

cg22190114

cg24870391

cg10883352

cg25527547

cg03977657

cg21624282

cg01835489

cg24835159

cg20556988

cg08458170

cg24147596

cg27622610

cg01580568

cg18462653

cg18223379

cg24833277

cg03332271

cg07908874

cg08886154

cg04711324

cg03116740

cg18623836

cg20324165

cg02833180

cg14333454

cg09847584

cg11206634

cg24276491

cg03684977

cg09936839

cg10145926

cg21201572

cg07711097

cg01119135

cg19686152

cg15422147

cg27341860

cg19831369

cg14851685

cg20797216

cg14209518

cg00757070

cg14404298

cg01441777

cg17233506

cg08214029

cg02237119

cg06848073

cg24134767

cg16609872

cg07705835

cg13300756

cg04329382

cg20484352

cg19258882

cg08124030

cg25426302

cg23499956

cg19759064

cg17471102

cg21602160

cg23579062

cg03003745

cg21250978

cg09307264

cg13439730

cg26672426

cg22889448

cg22580512

cg05245515

cg10052840

cg03752885

cg22131172

cg24855780

cg24620905

cg25946374

cg27324619

cg09152089

cg13928306

cg12603560

cg19923326

cg18053607

cg08578641

cg26143719

cg24765079

cg17826679

cg02293044

cg21663431

cg14519350

cg16509569

cg07380416

cg09902130

cg26285698

cg10590292

cg05751148

cg11600161

cg18384097

cg17936488

cg24497819

cg07973967

cg20792833

cg23612220

cg26158194

cg19663795

cg14145194

cg24545967

cg18621299

cg20425130

cg00447208

cg21898046

cg17771150

cg23506842

cg15258980

cg25671438

cg19252956

cg13821008

cg12177677

cg16068833

cg26149678

cg15691199

cg24088438

cg04275881

cg11804789

cg13470920

cg01348086

cg16749930

cg13273136

cg16777510

cg25866075

cg20994801

cg08818984

cg15679651

cg11201532

cg19005210

cg06855803

cg05989054

cg22407458

cg18611122

cg17199483

cg07376232

cg26928972

cg09682183

cg12108912

cg24450631

cg17709873

cg12836863

cg06144905

cg08804892

cg23352695

cg20018806

cg00168942

cg13634319

cg11098259

cg20070090

cg11484872

cg24625388

cg05501357

cg26267310

cg15840985

cg18940763

cg27365426

cg04797496

cg27562023

cg07086380

cg03548857

cg10934032

cg15387123

cg11203041

cg14114267

cg05246522

cg10037005

cg18908499

cg04301614

cg08585897

cg14451276

cg25229172

cg17998964

cg03294491

cg01001286

cg23213217

cg17527798

cg05130485

cg15645309

cg15261665

cg11304234

cg04915566

cg07935264

cg01280080

cg14448116

cg04228042

cg14902389

cg08529852

cg11761535

cg12564453

cg11998307

cg13354523

cg00597076

cg18493147

cg06910100

cg04995717

cg15127733

cg22844623

cg18533225

cg05064181

cg10275770

cg19537511

cg20967028

cg21012874

cg11398517

cg12876594

cg21142272

cg19368582

cg26245202

cg24664957

cg10157098

cg10061138

cg02254407

cg19242268

cg05046097

cg01169778

cg09243021

cg08779777

cg25902889

cg03636183

cg05050341

cg23679724

cg19731122

cg18771300

cg15407570

cg13797282

cg03483626

cg11136562

cg11484576

cg10569414

cg14289461

cg12456510

cg04123507

cg16501028

cg06003187

cg00350478

cg09299388

cg07039362

cg07613153

cg13412615

cg24919884

cg15903395

cg18552413

cg18153060

cg23444894

cg11939496

cg17983307

cg25363317

cg20647137

cg14269477

cg12032049

cg06403553

cg00601486

cg12200412

cg01087382

cg16666160

cg11812202

cg11481351

cg04498511

cg16581199

cg01671881

cg22077553

cg23514672

cg25093045

cg11465372

cg02423618

cg17561452

cg16084788

cg04618528

cg24030627

cg07830847

cg10377274

cg25141674

cg20485165

cg11234457

cg00745543

cg23749046

cg22988566

cg25737664

cg15554401

cg05260966

cg22445920

cg01469547

cg26628847

cg11113534

cg02807948

cg06849477

cg24457403

cg24697329

cg08203715

cg19370451

cg15481539

cg07753583

cg13379763

cg02737335

cg20488657

cg11706111

cg07879977

cg20543571

cg23382741

cg23767977

cg27108154

cg07478122

cg24024214

cg21256656

cg26504906

cg20661303

cg07728874

cg03112869

cg09995854

cg07947016

cg00510787

cg23865698

cg19324627

cg01869233

cg23627134

cg09747578

cg18022926

cg04081402

cg07165793

cg24293567

cg08012287

cg00201234

cg25288155

cg20664201

cg06821120

cg01110312

cg17602451

cg15195412

cg18015044

cg06980053

cg26561254

cg13755535

cg26608332

cg19815139

cg01138020

cg12228229

cg25827112

cg24691453

cg02735486

cg05656180

cg11432797

cg20725021

cg13904968

cg05163057

cg17974185

cg04008913

cg24269276
